# Local DEA app: Saving lives with accessible and well-located automated external defibrillators

**DOI:** 10.1371/journal.pone.0318065

**Published:** 2025-02-26

**Authors:** Giovanna Natsumi Eiri, Hudson Rogerio Proença Júnior, Miyoko Massago, Vinicius Lopes Giacomin, Karoline Harummy Romero Moriya, Mateus de Amorim Aboboreira, Felipe Hideaki Ueda, Aline Cardoso Machado, Júlia Loverde Gabella, Marcos Rogério Bitencour, Sanderland José Tavares Gurgel, João Ricardo Nickenig Vissoci, Heloise Manica Paris Teixeira, Luciano de Andrade

**Affiliations:** 1 Department of Medicine, State University of Maringá, Maringá, Paraná, Brazil; 2 Department of Informatics, State University of Maringá, Maringá, Paraná, Brazil; 3 Postgraduate Program in Health Sciences, State University of Maringá, Maringá, Paraná, Brazil; 4 Postgraduate Program in Management, Technology and Innovation in Urgency and Emergency, State University of Maringá, Maringá, Paraná, Brazil; 5 Department of Emergency Medicine, Duke University School of Medicine, Durham, North Carolina, United States of America; Jordan University of Science and Technology, JORDAN

## Abstract

**Purpose:**

Developing technological solutions to reduce care delay and mortality in out-of-hospital cardiac arrest (OHCA) cases is a challenge both in Brazil and worldwide. The aim of this study was to develop and validate a geolocation application for locating automated external defibrillators (AEDs) in the urban area of a medium-sized city in southern Brazil.

**Methods:**

A mobile application was developed to locate AEDs in public places, indicating the best route to the nearest AED with an intuitive design for emergency use. The app provides information on cardiopulmonary resuscitation (CPR), initial care, and AED use. The research steps included a literature review on the double diamond design process, prototyping, expert validation, and simulation tests.

**Results:**

The Local DEA app, developed for Android and iOS, offers features such as route calculation to the nearest AED and emergency services. In an evaluation conducted by 30 experts, the app was well accepted for its ease of use and efficiency (Cronbach’s alpha = 0.92). Simulation tests demonstrated that the strategic installation of accessible and well-located AEDs improves coverage and significantly reduces travel time to emergency care as compared with ambulance travel times (*p* < 0.001).

**Conclusion:**

The Local DEA app is a promising tool for reducing mortality in OHCA, enabling AED geolocation, use instructions, and CPR guidelines. When combined with the strategic placement of public-access AEDs, the application can increase the likelihood of survival in OHCA cases.

## Introduction

Out-of-hospital cardiac arrest (OHCA) is a persistent global challenge affecting both developed and developing countries. According to Rajan et al. [1], 60% to 80% of deaths from cardiorespiratory arrest occur in out-of-hospital settings. Mortality in these sudden events is influenced by several factors, such as underlying clinical causes, the response time of pre-hospital care services, the quality of cardiopulmonary resuscitation (CPR), the presence of CPR-trained lay rescuers, and the availability of automated external defibrillators (AEDs) [2]. OHCA reversal requires prompt care by healthcare professionals and/or laypersons. AEDs are essential for increasing the chances of survival of OHCA victims. In this context, ’success rate’ refers specifically to the proportion of patients who survive to hospital discharge with favorable neurological outcomes, a critical indicator of the effectiveness of interventions in out-of-hospital cardiac arrest. Early defibrillation plays a pivotal role in achieving these outcomes; when performed within 3 to 5 minutes of cardiac arrest, survival rates can reach 50% to 70% [3]. This underscores the importance of timely access to automated external defibrillators (AEDs) in improving patient survival.

Despite the growth of AED programs (e.g. in Japan, Sweden and the USA), many limitations to early defibrillation in cardiac arrest persist. A public AED is used in only a small percentage of OHCAs. Barriers to AED use include inaccessibility, lack of awareness, and negative perceptions [4]. AED accessibility is often limited, either by a scarcity of devices or a lack of knowledge among the population about their location and use [5]. For instance, many establishments that have AEDs are not open 24 h a day, which makes these devices inaccessible during the night and on weekends. As OHCA can occur at any time, restricting access to AED devices to regular opening hours impacts their use by the population [6].

In Europe, public access defibrillation (PAD) programs have played a critical role in reducing mortality from out-of-hospital cardiac arrests (OHCA). These initiatives, supported by organizations like the European Resuscitation Council and the International Liaison Committee on Resuscitation (ILCOR), have focused on strategic AED placements in high-traffic areas such as airports, stadiums, and public transport hubs. Alongside these efforts, extensive public education campaigns have aimed to increase awareness and confidence in using AEDs. Studies show that these measures have improved survival rates, though challenges such as underutilization and access inequalities persist. These programs illustrate the importance of leveraging both technology and public engagement to address life-threatening emergencies effectively [7]. Poland exemplifies significant efforts to address out-of-hospital cardiac arrests (OHCA) through public access defibrillation (PAD) initiatives. While metropolitan areas like Warsaw and Kraków have advanced in deploying automated external defibrillators (AEDs) and raising public awareness, rural regions still face critical gaps in access and education. Innovative programs such as ’Impulse of Life’ highlight the country’s commitment to expanding AED availability and integrating community-driven strategies. Despite systemic challenges, Poland’s progress demonstrates how localized approaches can adapt broader European PAD strategies to diverse sociodemographic needs [8, 9].

In Brazil, another relevant limitation is the lack of physical maps or geolocation applications for locating AEDs in public places. Moreover, the few available AEDs are usually located in private spaces. Most of these devices were purchased from online distributors, either wholesale or individually for private use. AED registration is the owner’s responsibility, and the registration process varies by region. Therefore, the lack of centralized registration data represents a barrier to the mapping and surveillance of these devices, impacting accessibility [10]. Additionally, the locations where AEDs are installed typically do not correspond to the areas where cardiac arrests are most frequent [11]. This lack of alignment between AED locations and high-risk areas contributes to the underutilization of the devices, which, combined with the lack of lay training and concerns about legal responsibilities, hinders an effective and rapid response in emergency cases [4].

International studies have used georeferencing data as an additional tool to improve OHCA care [12, 13]. Likewise, there is a growing availability of health-related mobile applications that can empower individuals to respond to OHCA events. To the best of our knowledge, no comprehensive studies have been conducted in Brazil on the design, development, and validation of a mobile application that uses geospatial resources for the location of AED devices. This study aimed to fill this gap by developing and validating a geolocation application for locating AEDs in the urban area of a medium-sized municipality in northern Paraná State, Brazil.

## Materials and methods

### Study design and location

The mobile app was developed following the four steps of the double diamond design process within the design thinking and innovation framework: discover, define, develop, and deliver [14]. Subsequently, the application was validated by a group of experts.

The study was conducted in Maringá, Paraná State, southern Brazil. Maringá has an area of 487.012 km^2^ and a population of 409.657 inhabitants (2022 data), corresponding to a population density of 841.16 people/km^2^. The ≥ 60 years age group comprises 75.004 individuals, that is, 18.3% of the total population [15]. Geographically, the municipality is divided into 536 census tracts that correspond to territorial units comprising approximately 250 to 300 households, depending on population density, 416 of which are located in the urban area [15].

### Discovery: OHCA care and emergency service activation in Brazil

In the first step, the double diamond design process guides the discovery of the real problem [14]. Empirical observations are confronted with the available literature to gain a better understanding of the problem and formulate hypotheses to define the target product [16]. The assumption of this study was the following: laypeople do not know where to search for an AED when faced with a victim of cardiac arrest on a public road. This assumption combined the project scope and kick-off point, serving as a starting point for app development. However, it was first necessary to understand the provision of OHCA care in Brazil.

**Fig 1:**
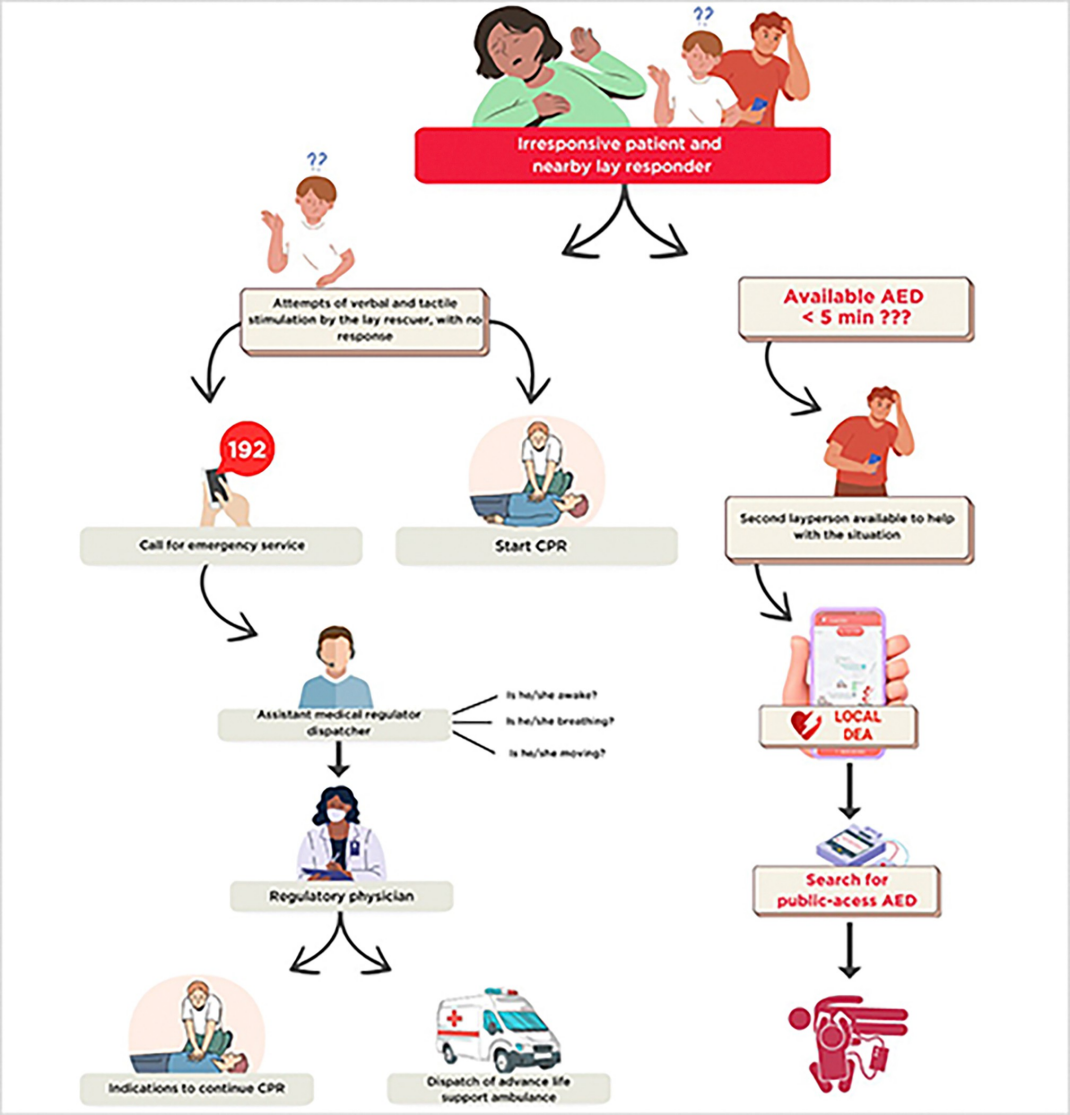
Flowchart describing the steps in providing care for victims of out-of-hospital cardiac arrest. AED, automated external defibrillator; CPR, cardiopulmonary resuscitation.

In an OHCA situation, the victim is typically found falling on the ground or unresponsive on a public road or at home. The person who identified the situation (lay responder) makes attempts at verbal and tactile stimulation. Upon not getting any response, the responder should immediately call the mobile emergency care service, which in Brazil is known as SAMU [1] (Fig 1).

At the SAMU call center, calls are answered by an emergency call technician, who collects information on the victim’s location, condition, and, whenever possible, medical data. The collection of basic information should be concluded in the shortest time possible. Emergency call technicians are trained with service protocols to identify phrases indicative of a potentially severe semiology syndrome, such as OHCA. Examples include: "I found him/her passed out," "he/she is purple," "he/she is not breathing," and "he/she is not moving" [17].

After this initial screening, the emergency call technician forwards the call to the regulatory physician, who performs a detailed assessment of the situation using specific questions directed to the caller. In OHCA cases, questions aim to assess the level of consciousness, respiratory status, and circulation (vital signs). The procedures are tailored to the specific emergency. For example, in the case of cardiac arrest, the regulatory physician prioritizes rapid identification of the event, often based on key indicators such as the absence of a pulse and unresponsiveness. After categorizing the situation as cardiac arrest, the regulatory physician initiates the dispatch of the nearest available resources, preferably an advanced life support ambulance, while guiding the caller to begin basic life support measures, such as chest compressions and the use of an automated external defibrillator (AED), if accessible. The regulatory physician should remain on the line to provide ongoing instructions until the emergency team arrives at the scene [17]. Meanwhile, the physician instructs the caller to start CPR.

The ideal in such events would be for another individual (bystander) to be present at the scene, know the location of a nearby AED device (roundtrip travel time < 5 min), and be able to use the device. Once the AED is attached to the victim, it identifies whether the heart rhythm is shockable and, if so, delivers the electric shock while SAMU does not arrive. Having prior knowledge about the location of AEDs saves valuable time, allowing defibrillation to be initiated as soon as possible, which is crucial to increase the victim’s chances of survival [17]. However, most of the population does not know where public-access AEDs are located. It should be noted that there is a consensus in the literature that cardiac arrest, regardless of whether it occurs inside or outside the hospital, is a time-dependent event. In other words, rapid high-quality care is essential to improve the outcome [18]. Thus, it is essential to know not only the location of AEDs distributed throughout the city but also the fastest route to the nearest equipment.

### Define: Guiding users to the nearest AED

In the definition step, the feasibility of the proposed solution is analyzed in light of available resources. Here, the solution to facilitate the location of AEDs by the population was to design a geolocation app. This app would allow users to quickly find the nearest AED, optimizing response time in emergency situations and potentially saving lives.

Several studies endorse the main difficulties reported by prehospital care providers in providing timely treatment for OHCA cases. These difficulties include a lack of widespread knowledge among the general population about the location of public-access AEDs [5, 6] and the recommended procedures for CPR provision by laypeople [4, 6]. Adding to this situation, the arrival time of the prehospital care team at the scene is often longer than the recommended for providing initial care in OHCA cases [12].

There are several mobile applications that provide interactive maps showing AED locations in the United States and European and Asian countries [19–25]. Some examples are described in S1 File. These applications were assessed for functionality and potential weaknesses. Each application was downloaded and examined manually and individually to identify the most relevant points of each technological solution.

Analysis of the above-mentioned applications revealed several limitations that could compromise their effectiveness and usefulness. Most applications do not offer a feature that calculates and suggests the shortest route for different means of transport, such as car, bicycle, or walking. This limitation precludes users from receiving optimized guidance for their specific mode of transportation, potentially increasing travel time and decreasing the victim’s chances of survival. Additionally, it was noted that the applications lacked a screen for users to send suggestions and feedback directly to developers. A feedback screen would allow users to easily share their ideas and help improve the app. Another point worthy of mention was the lack of a feature that provides quick and practical access to an updated registry of hospitals and emergency care units (UPAs), including the distance and travel time to these locations. The absence of this information could lead to delays in receiving medical care in emergency situations.

As part of the definition step, an active search for AEDs was carried out in public establishments with high foot traffic. This procedure was necessary because of the lack of available information from municipal health agencies regarding the locations of AED devices. The search found 5 AEDs in the city, which were placed on the App. Based on the collected information and the gaps identified in existing applications, an innovative application was designed to offer a comprehensive and efficient solution to the problem at hand. Key features of the app include:

1. Global positioning system (GPS) location of AEDs. When the user opens the app with the GPS turned on, they can quickly view the location of nearby AEDs.
2. Search for the nearest AED. The app features a red button at the center of the screen named "Search for AED." When the user clicks this button, the application automatically calculates and displays the closest AED to the user’s current location based on smartphone GPS data.
3. Route calculation and travel time. The application not only displays the location of the nearest AED but also calculates and shows the distance and estimated time to reach it using various means of transportation (on foot, by car, or by bicycle). This feature allows users to choose the fastest and most efficient route to an AED, depending on the circumstances and resources available at the moment.
4. AED use instructions: After locating and retrieving the AED, users can rely on the app to obtain clear instructions on how to use the device. Instructions are provided via audio by the AED device, guiding the user step by step on how to apply electric shocks to the victim, if needed, until the arrival of SAMU.

### Development

Prototyping and app development were performed in the Dart programming language [26] using the Flutter framework [27]. Flutter, a user interface development kit, enables the creation of interfaces for Android and iOS devices using a single programming language (Dart). Dart features a syntax similar to other languages that adopt object orientation as the main programming paradigm, facilitating the development of applications in accordance with architectural standards for the software industry. The source-code editor VScode was used for code compilation and application development [28]. Architectural patterns and software design followed the procedures described by Robert Martin in his book Clean Architecture [29].

Geolocation services use the HERE Maps tool [30]. Geolocation is a technological feature that allows determining the geographical position of a device using a coordinate system. Geographic coordinates can be obtained by different methods, the best-known of which is GPS. GPS coordinates are derived from data provided by satellites, which process this information and use triangulation methods to determine the approximate location of the receiving object.

It is possible to access GPS geolocation on mobile devices through map services. These services offer a graphical and communication application programming interface (API) that enables the use of various GPS features. A well-known example is Google Maps. Google Maps can be used to obtain a map interface, interact with it, edit it by adding coordinates and reference points, and save locations on the map. The communication API [30], on the other hand, offers features such as route calculation and determination of the shortest path to a specified location, which are among the key functionalities of the proposed application.

The requirements survey was based on the general requirements of the project, user interface and interaction analysis, and adaptation of related works with similar objectives. The developed application was called Local DEA. It facilitates the location of AEDs and other medical emergency services, allows the user to identify the shortest route to the selected service, provides information about AEDs and cardiac arrest, and includes a feature for the manual registering of emergency services.

In the design stage, the free versions of Canva [31] and Figma [32] were used to construct screens, texts, and visual elements. Graphic resources available for free on the Freepik website [33] were also used. The images in the illustrative guide with CPR instructions were created by a product designer.

The application was registered with the Brazilian National Institute of Industrial Property (INPI) in partnership with the Technological Innovation Center (NIT) at the State University of Maringá (UEM), Paraná, Brazil, under the intellectual property number BR 512022002300–8.

### Delivery: Expert validation

Experts were randomly selected to participate in the validation of the developed application, including doctors, nurses, and nursing technicians who worked in emergency care or intensive care units at the university hospital in Maringá, Paraná State, Brazil, during the research period (December 2022 and January 2023). A total of 30 evaluators were included in the study after signing a voluntary participation agreement and an informed consent form approved by the Ethics Committee.

At the time of the evaluation, the research team presented the experts with a detailed scenario of OHCA. Then the experts received a smartphone with the application prototype installed and were instructed to test its functionality and usability under simulated conditions. After the demonstration and use of the application, each expert answered a questionnaire based on the Computer System Usability Questionnaire (CSUQ) (S2 File) [34]. The questionnaire was created using Google Forms.

CSUQ is a widely used usability questionnaire that assesses user satisfaction with the performance of computer systems, websites, software, and mobile applications. It is designed to evaluate several aspects, including overall satisfaction, which measures the user’s perception of system usability; efficiency, which refers to the system’s ability to assist users with tasks; utility, which assesses how well the system meets user needs; and interface quality, which evaluates the ease of use and graphical presentation of the interface [34]. The questionnaire contains 16 objective questions [34] rated on a 7-point Likert scale, where 1 corresponds to "strongly disagree" and 7 to "strongly agree." This rating system aligns with common practices in the literature, which suggests using scales with 5 to 9 options to evaluate usability and user satisfaction [35]. The reliability of the CSUQ questionnaire was confirmed by measuring the global Cronbach’s alpha coefficient. Subscale analysis was not conducted, given that the tool is validated and widely used.

### Simulation scenario

A small-scale spatial simulation was performed to examine the contribution of a geolocation application to the strategic placement of AEDs in the urban area of the studied municipality. Travel times to strategically installed AEDs were compared with ambulance travel times in emergency situations. The city center was selected for the simulation due to its high population density and presence of a nearby ambulance station. A total of 50 points were randomly plotted in the center region using the generated random points in the extension algorithm of QGIS software version 3,28, with each point representing potential OHCA occurrences. Two simulations were performed for each point. The first estimated the response time from the ambulance station, and the second modeled the AED position and the travel time to and from the AED device.

In the first step, the probability of an ambulance responding to simulated occurrences within 5 min was determined by creating a distance matrix using Valhalla plugin version 2.4.2, available as an extension to QGIS software. This matrix was used to calculate the travel time between the ambulance station and the scene, making it possible to determine the number of points attended within the predefined time limit.

In the second step, AED allocation was simulated using the maximum coverage location problem (MCLP). The analysis was conducted using the Gurobi Optimization application, available in Python language. MCLP is a mathematical problem that seeks to determine the most effective location for the installation of resources, in order to maximize the coverage of the desired points [36]. The simulation considered only pedestrian displacements to and from potential AED locations (2.5 min for each stretch). A standardized walking speed of 10 km/h was used for the creation of the distance matrix [37].

The candidate sites for AED installation were selected among public and non-residential spaces, identified mainly through the points of interest provided by the Maringá Institute for Research and Urban Planning (IPPLAM). The location of supermarkets and hypermarkets was obtained from the ICMS taxpayer registry of the State Revenue of Paraná, and those of long-term housing were identified through the official website of the municipality.

After completion of the two simulations, a comparative evaluation was carried out between the median times of ambulance response and bystander AED retrieval. The data were subjected to the Shapiro–Wilk test for assessment of the normality of distribution. Given that the assumptions of normality were not met, comparisons were performed using the Wilcoxon test.

### Ethical approval

The research was authorized by the Regional University Hospital of Maringá and approved by the Human Research Ethics Committee of the State University of Maringá (Protocol No. CAAE 58406922.7.0000.0104). Health professionals participating in this study were informed of the risks and benefits and confirmed their participation by signing an informed consent form between December 2022 and January 2023, as approved by the ethics committee. In compliance with data confidentiality and reliability laws, the information provided by participants was stored and processed in accordance with Law No. 13.709/2018 (Brazilian General Data Protection Law).

## Results and discussion

### Design thinking

The Local DEA app was created following the cyclical process of design thinking and the double diamond method [14]. The name of the app originates from the combination of the radical "local" from the word "locate" and "DEA," which is the acronym for AED in Portuguese (*desfibrilador automático externo*).

### Requirements gathering: Discovery and define

Requirements gathering was achieved through a collaborative workshop with the project team, composed of professionals and students in the areas of Health and Computer Science. During the meetings, brainstorming was used to align the expected functionalities of the product with technical feasibility, following the empathetic and user-centered model proposed by design thinking. On the basis of key precepts, prototyping was used to explore and identify the requirements interactively. Furthermore, similar systems proposed in related studies were analyzed.

Once the discovery phase was completed, the team initiated the phase of definition. The following functional requirements of the app were determined: (1) identify the shortest route to an AED for different means of transport, (2) provide visual information through Google Maps, (3) identify the shortest route to an emergency service, (4) register and consult emergency service information, (5) place a quick call to emergency services, (6) provide a channel for user suggestions and frequently asked questions, and (7) provide a guide on how to perform CPR.

The following non-functional requirements were defined: (1) reliability, the app must ensure user authentication and provide answers consistent with real situations; (2) privacy, user data must be confidential; (3) performance, the app must function quickly on any mobile device and support multi-user processing, that is, simultaneous use of the app by several users; (4) usability, the app must have a simple and intuitive interface for easy use in emergency situations; (5) portability, the app must be compatible with Android and iOS systems.

The functional and non-functional requirements were documented in a study case model and made available to the development team.

### Development

**Fig 2:**
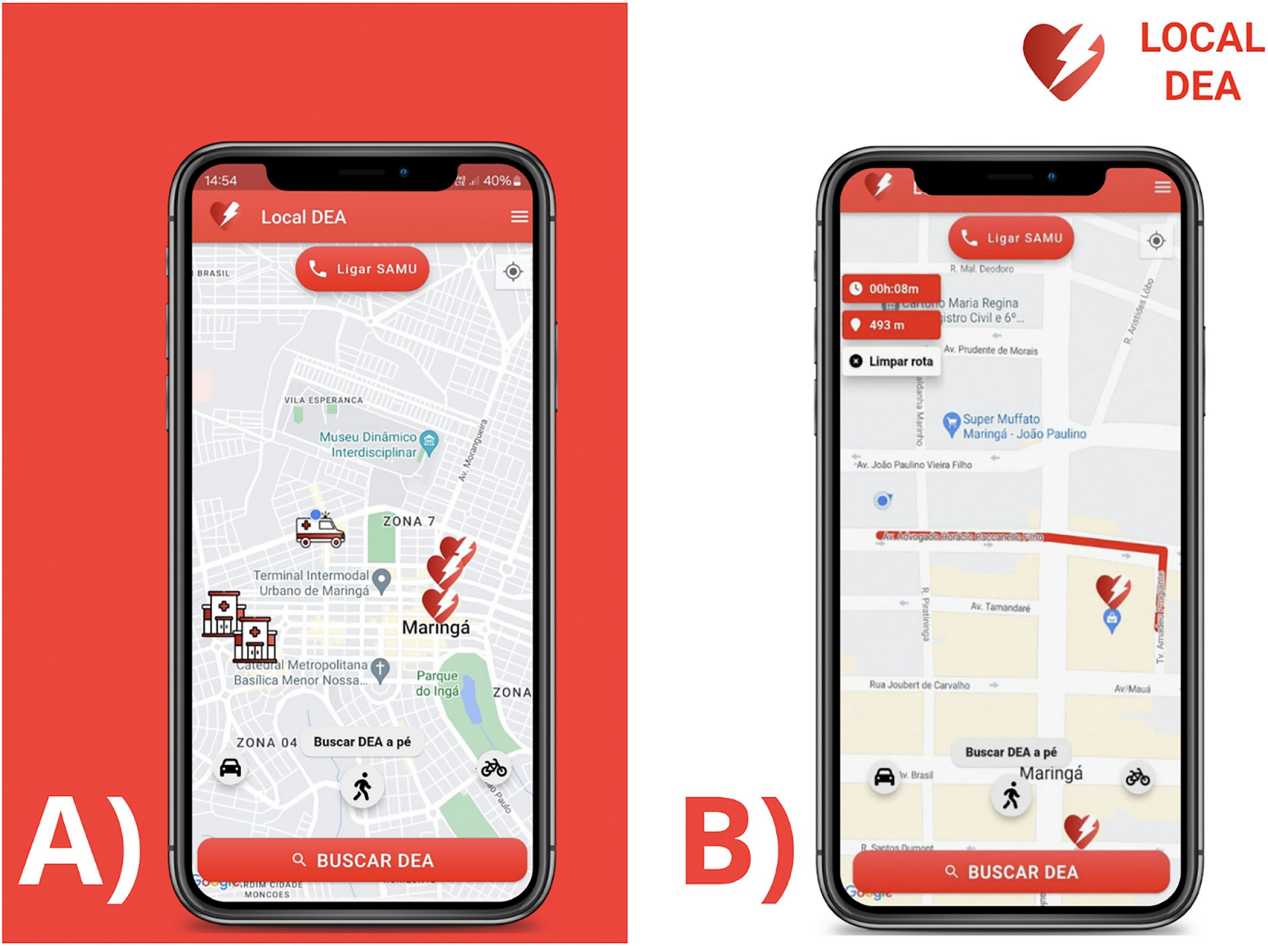
**A** Home screen of the Local DEA app. The image displays a map with the location of the nearest automated external defibrillators (AEDs), hospitals, and ambulance bases. **B** Example of the shortest route to an AED by walking.

The app was developed for devices running Android version 8.0 (Oreo). The Flutter framework was used for compatibility with iOS devices. On the main screen, the Local DEA app displays a map showing the user’s current location, as well as that of nearby AEDs and emergency medical services (hospitals, UPAs, SAMU bases) via geolocation. Its main function is to calculate the shortest route to the nearest AED, providing detailed information of the route and estimated time (minutes) and distance (meters) (Fig 2A). The route is calculated considering the means of transport selected by the user (e.g., on foot, by vehicle, or by bicycle) (Fig 2B).

The home screen of the app is displayed in Fig 2A. On this screen, users may contact emergency services by clicking the red button "Call SAMU," located in the upper middle part of the screen. The user’s location can be centered by clicking on a button located on the top right side of the screen. One can also adjust the orientation of the map to suit the geographic north by clicking the button in the upper left corner (compass icon). The three lower icons represent means of transport (car, foot, and bicycle), and the route to the nearest AED is calculated by clicking on the red button with the phrase "Search for AED," preceded by an icon of a magnifying glass.

Fig 2B shows the steps for calculating and visualizing the route to the nearest AED. The image displays an 8-min walking route. In the center of the screen, a red polyline indicates the path to be followed. In the upper left corner, the duration and total distance of the route are displayed. A red heart with a white flash marks the position of the AED. The lower part of the screen displays the three transport options available, with the current selection highlighted.

The route may vary depending on the location of the user and the type of transport chosen. In Fig 2B, for example, the route was calculated for walking; therefore, the traffic patterns of Maringá were not taken into account, such as the direction of travel of some streets. Route calculation also takes into account local traffic; thus, the shortest distance is not always the recommended route. Health agents authorized by the municipality’s Secretary of Health can register establishments with AEDs, informing their location, name, and phone number. These registered services are displayed to all users on the integrated map of the app.

**Fig 3:**
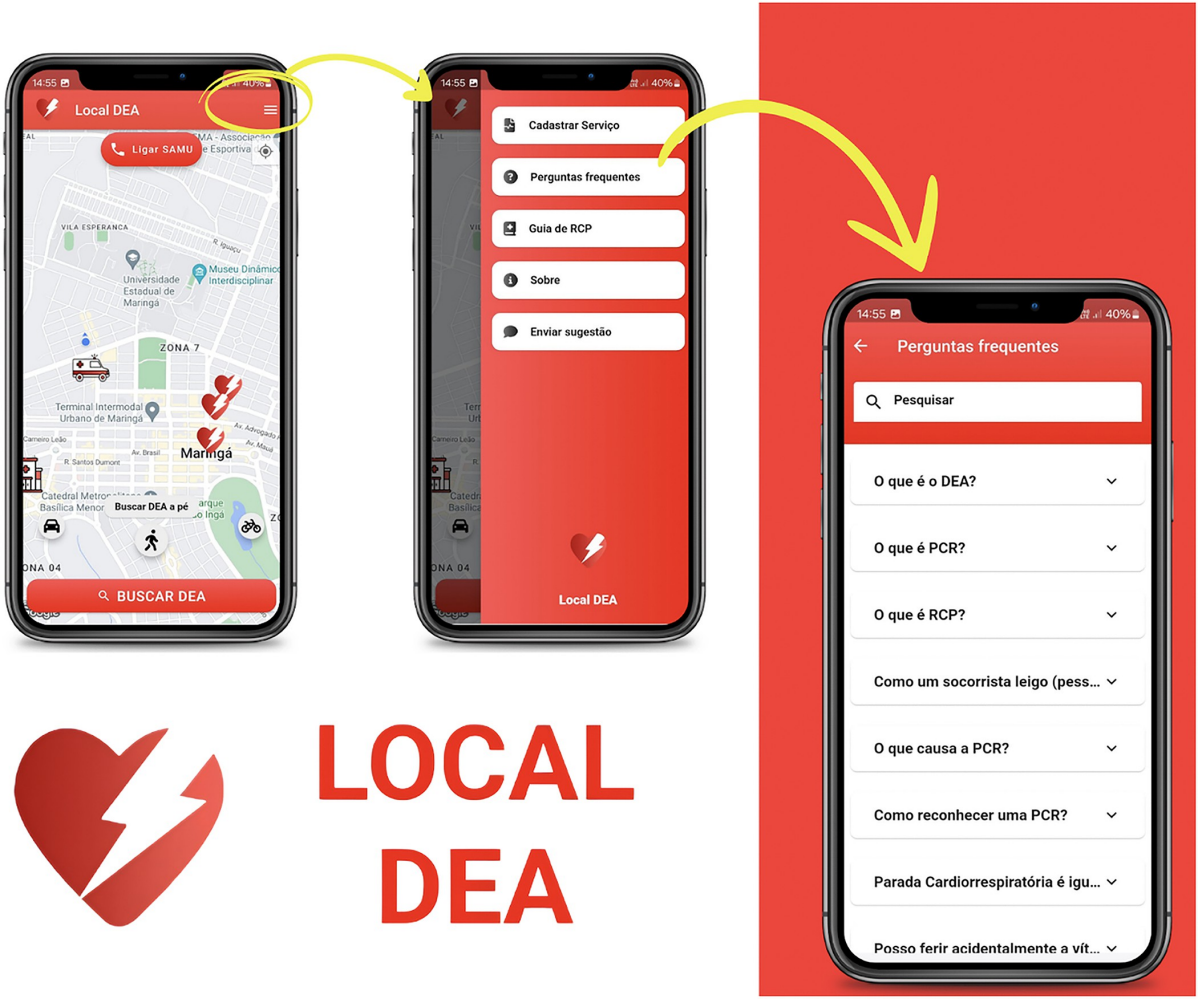
Frequently asked questions tab of the Local DEA app.

It is also noteworthy that the app presents a list of frequently asked questions regarding AED use, OHCA, and CPR. This section is intended to contribute to the education of users on the subject, resolving their doubts and curiosities (Fig 3). Users may also submit suggestions to the development team, contributing to enhancing customer experience and promoting constant improvement. The button can be accessed by any user on the app menu. It redirects users to their preferred email application with the address of the development team.

**Fig 4:**
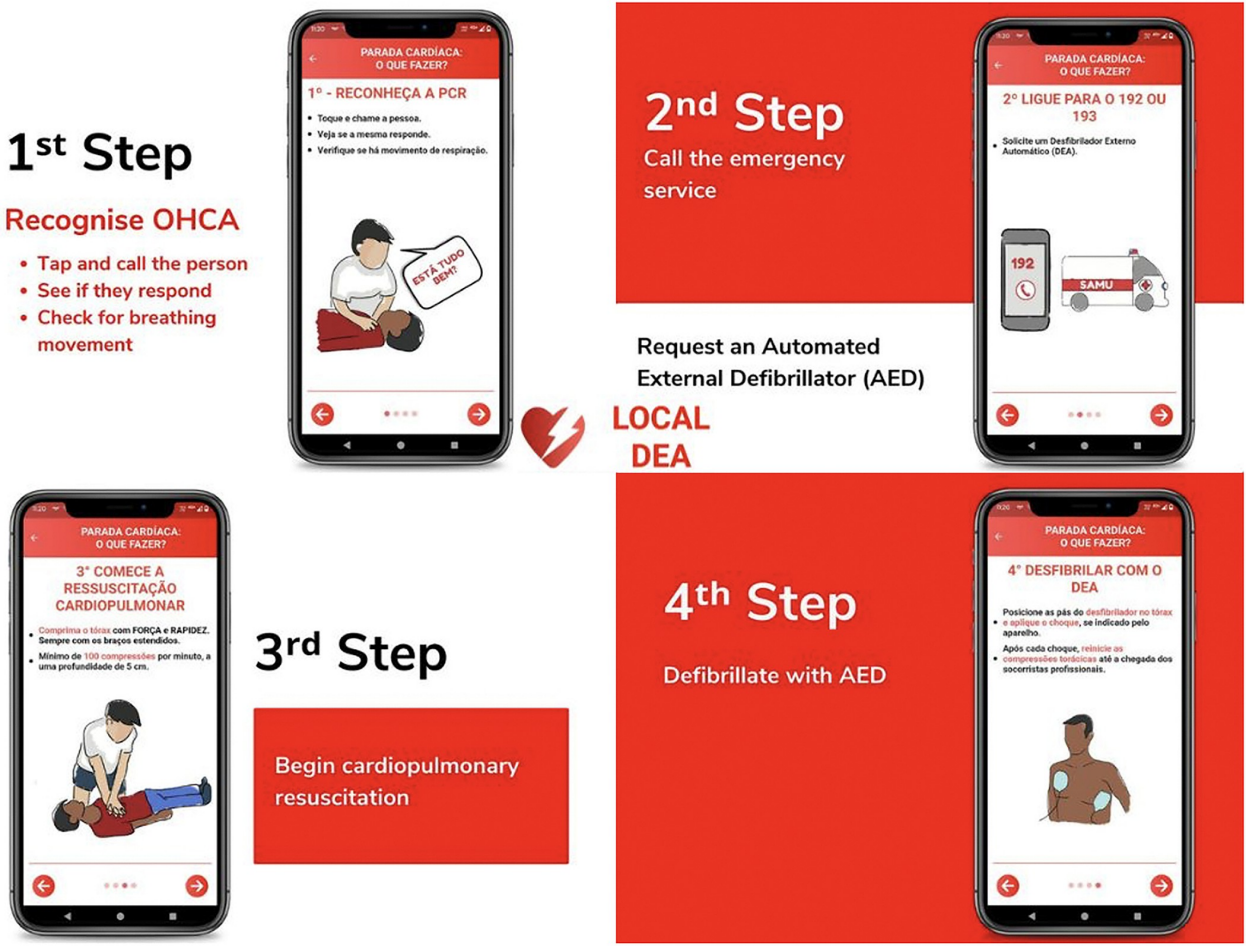
Illustrated guide of the Local DEA app on how to provide initial care for out-of-hospital cardiorespiratory arrest (OHCA).

As a secondary and informative feature, the app has a screen presenting an illustrated guide on how to provide initial care for OHCA. Such care can be performed by a layperson, according to guidance from the American Heart Association [38]. The app also demonstrates when and how to use an AED (Fig 4). The procedure is divided into four steps.

In the first step, it is explained how to recognize an OHCA by first touching and calling the person, to monitor their response to the stimuli, and checking for breathing movements. Then, the user is guided to call emergency services (192 or 193) to request an AED. In view of the possible difficulty in thinking clearly during urgent situations, the app has the added function of dialing the SAMU number, preventing call delay due to forgetfulness. In the third step, the layperson is recommended to perform CPR. An illustrative guide is provided (Fig 4), containing instructions about the correct CPR technique, including the ergonomics, strength, and speed of movements. Finally, in the fourth step, the layperson is instructed to use an AED. An illustration explaining how to use an AED is provided (Fig 4), with instructions on the correct positioning of defibrillator pads, detection of shockable rhythms, and the need to intersperse CPR with AED. With these guides, the layperson can provide care until the arrival of the professional emergency team.

**Fig 5:**
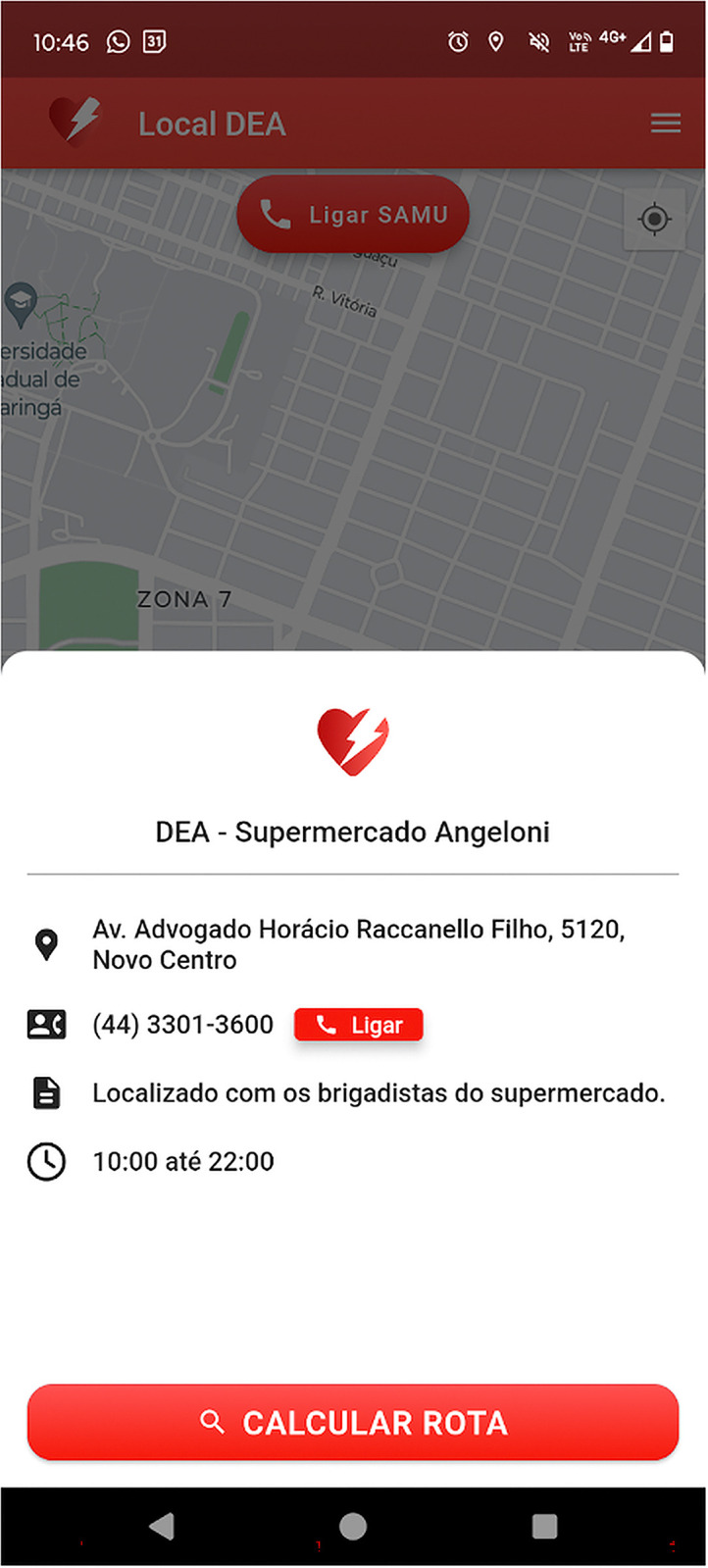
Panel of the Local DEA app showing information about an automated external defibrillator. The image includes the location, telephone number, available healthcare staff, and opening hours of the establishment.

A functional and important feature of the app is the possibility of viewing specific information about AEDs (Fig 5). The address, responsible team, phone number, and opening hours of the establishment housing the AED are provided. The app gives users the possibility of directly calling the establishment, allowing them to quickly check whether the place is open. At the bottom of the information panel, users can easily calculate the shortest route to the AED.

Overall, the main functions of the Local DEA app are the following: calculation of the shortest route and time to the nearest AED according to the chosen means of transport; location of public-access AEDs housed in public or private establishments; location of emergency services with AEDs (e.g., UPA units, hospitals, and SAMU bases); visualization of contact information of emergency services and establishments with AEDs; registration of establishments with AEDs by users; server authentication; user suggestions to the app development team via email; placing an emergency call to SAMU; placing a call to the AED establishment and emergency medical service; identification of the development team, description of the application, and contact details; frequently asked questions section regarding OHCA and AED; and CPR guide for lay people.

### Expert evaluation of the prototype

The Local DEA app was evaluated by 30 experts at the University Hospital of Maringá. Of these, 46.7% were aged 20–29 years, 33.3% were aged 30–39 years, and 20% were aged 40–49 years. The majority of the sample were women (56.7%). Regarding schooling, 76.7% had completed higher education or held a post-graduate degree, whereas 23.3% had incomplete technical or higher education. The area of expertise of all participants was Health, with 90% working in medicine, nursing, or physiotherapy. Most participants (80%) reported having experience using apps.

**Fig 6:**
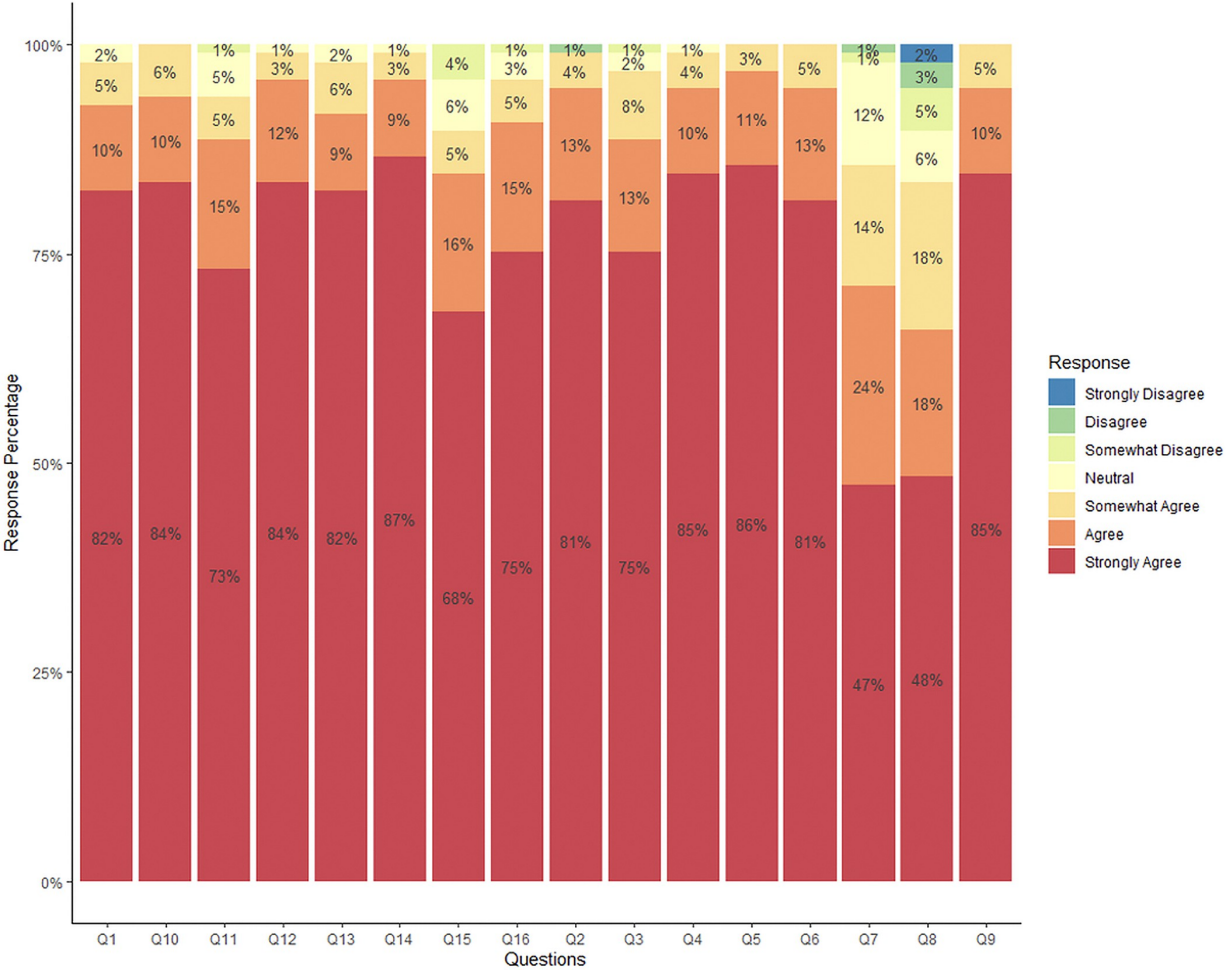
Distribution of responses to the Computer System Usability Questionnaire rated on a Likert scale.

Most of the items of the CSUQ instrument received neutral or positive results (Q1, Q4, Q5, Q6, Q9, Q10, Q11, Q12, Q13, and Q14) (Fig 6). However, Q2 (it is simple to use the app), Q3 (I can easily remember how to use the app), Q7 (I can easily and quickly recover from mistakes when using the app), Q8 (it would be easy to become skilled at using the app), Q11 (the app meets my needs), Q15 (the app does everything I would expect it to do), and Q16 (the app works the way I want it to) received some negative answers. More specifically, 1% of respondents strongly disagreed with Q2; 1% disagreed with Q3, Q11, and Q16; 2% disagreed or strongly disagreed with Q7; 10% somewhat disagreed, disagreed, or strongly disagreed with Q8; and 4% disagreed with Q15. The results indicate that the app was well-rated in terms of ease of use and functionality, but some specific areas require improvement, related to the simplicity of use, memorization, error recovery, and ease of becoming skilled in using the application.

Result reliability was assessed by using the global Cronbach’s alpha, which was found to be 0.92. This indicates excellent internal consistency, which reflects the reliability of the scale used.

### Simulation results

**Fig 7:**
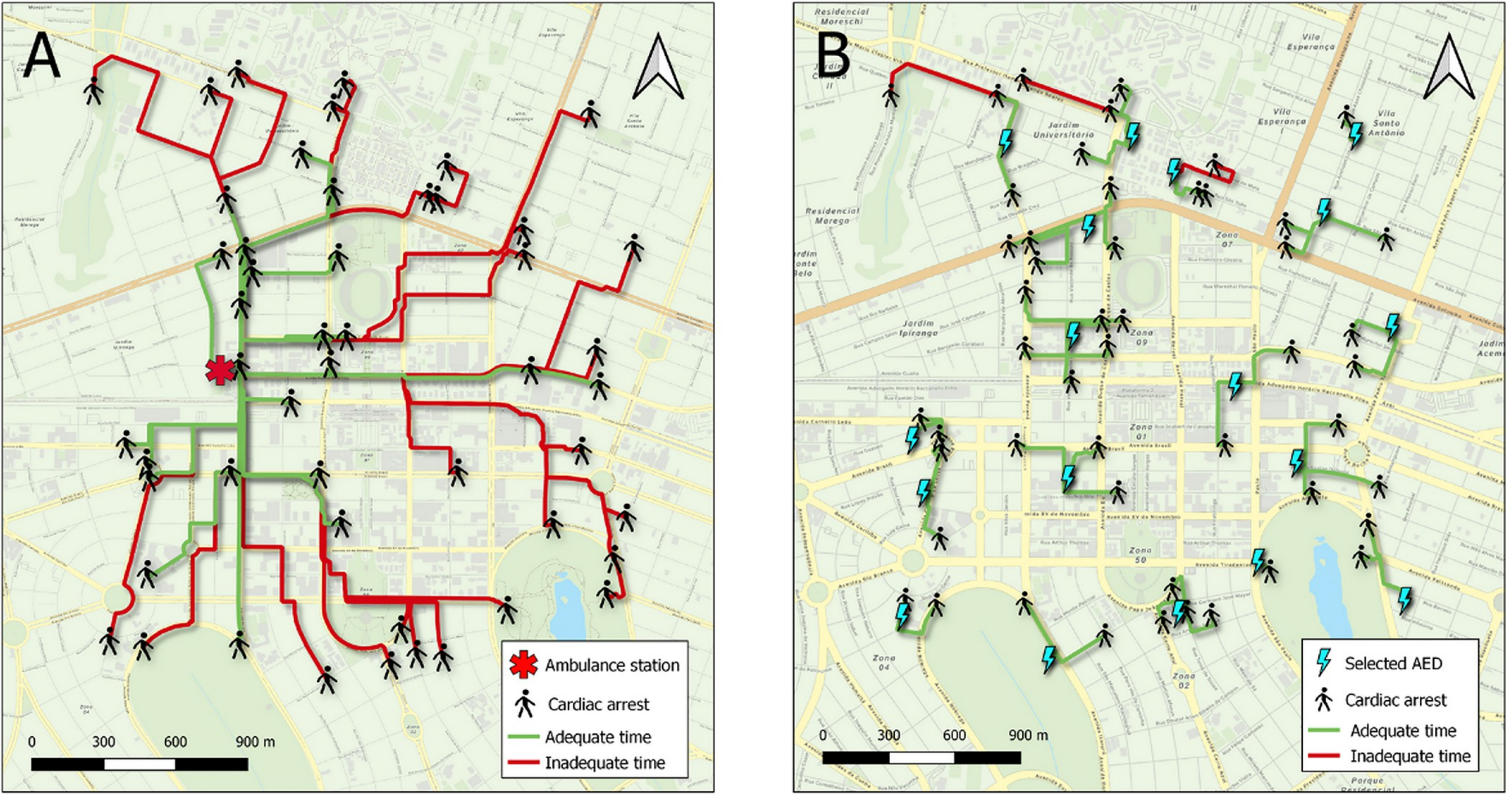
Simulation of an ambulance travel time to occurrence sites of out-of-hospital cardiorespiratory arrest and B roundtrip travel time to automated external defibrillators (AEDs) by bystanders.

In the simulation based on ambulance travel times from the central ambulance station, it was found that only 23 of the 50 OHCA events could be attended within the 300 s interval, with a mean travel time of 309 s (95% CI = 308s, 311s) and a median time of 327 s. MCLP optimization identified 18 sites in the central area of the municipality for AED allocation (Fig 7). With this, 47 of the 50 OHCA events could be attended within 300 s, with a mean response time of 223 s (95% CI = 223s, 225s) and a median of 206 s.

**Fig 8:**
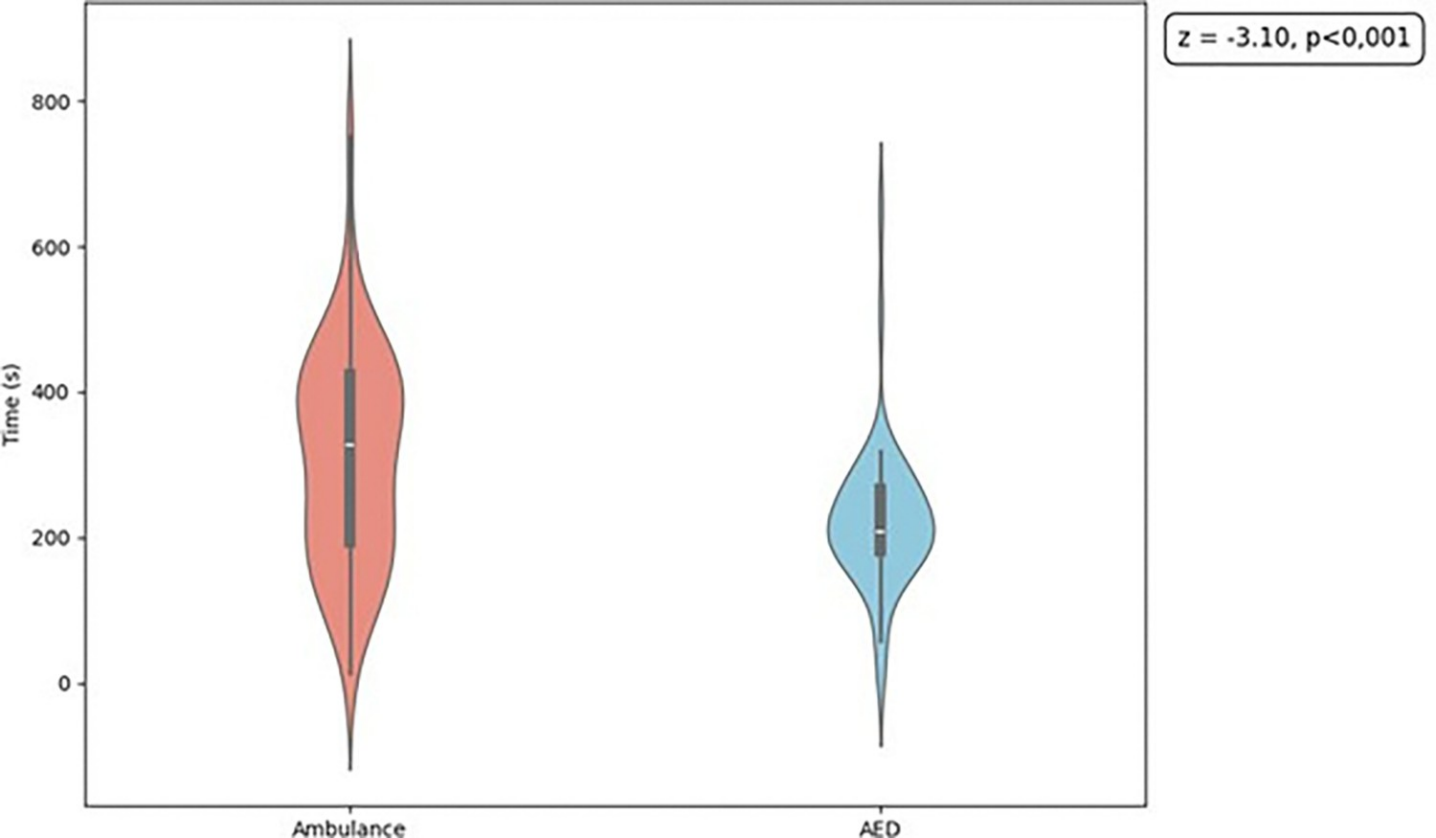
Violin plot. The plot compares ambulance travel times to occurrence sites of out-of-hospital cardiorespiratory arrest and bystander roundtrip travel times to automated external defibrillators (AEDs).

Comparative analysis of medians using the Wilcoxon test resulted in a *z* value of −3.105 (*p* < 0.001) (Fig 8). This finding reveals significant differences between medians, demonstrating that strategic allocation of AEDs (selected by the MCLP model) would be more effective for OHCA emergencies in terms of coverage and travel time compared with ambulance travel times.

## Discussion

Reducing OHCA response time and mortality remains a great challenge in Brazil and many countries around the world. In view of the need to act quickly in such cases, this study developed a mobile application to assist lay people in locating the nearest AED or emergency medical service, optimizing response times and increasing the chances of survival. To the best of our knowledge, there are no studies that address the design, development, and validation of an app using geospatial resources to locate AEDs in Brazil.

The main feature of the Local DEA app is the geolocation of AEDs in a medium-sized municipality, calculating the time and shortest route to the device. The app could positively impact the outcome of OHCA victims by offering the quickest and most convenient way to locate AEDs. AED accessibility and knowledge of their location are real difficulties in the care of OHCA [39]. The app also has important secondary features, such as an illustrated guide on how to provide initial care for a patient experiencing OHCA. Such information is particularly important for people without experience in this situation. The app provides a frequently asked questions section on AED use, which may assist bystanders during an OHCA event. These resources are fundamental, given that many people fear that the device may pose risks to the operator, find it to have a complex application technique, and have little or no knowledge of its correct use [40]. Furthermore, the app provides specific information about AED location, such as the address and telephone number of the establishment, facilitating contact and clarification about the device’s availability. The AEDs are retrieved from a server using Firebase. It’s cloud-based, and every time the app is opened, it fetches the latest data from the server. Users will not need to reinstall the app, as the data is fetched online from the server. Whenever an AED is added, users are automatically updated.

In the present study, CSUQ was used to evaluate the usability of the app by specialists. Most answers were positive, evidencing the app’s ease of use and suggesting that it is intuitive and effective for real situations. Furthermore, the instrument had excellent internal consistency, as shown by the global Cronbach’s alpha value (0.92). This result indicates a strong agreement between participants’ responses, reinforcing the reliability of the data and the robustness of CSUQ in assessing the usability of the app.

Following validation by experts, a test simulation was conducted, revealing the importance and effectiveness of the app. In the simulation, 47 of the 50 OHCA events were covered by AEDs within 5 min (for this simulation, it was assumed that lay first responders traveled at a running speed of 10 km/h), showing excellent coverage. In the same time interval, the ambulance station attended only 23 OHCA cases. This result confirms that, given the strategic allocation of AEDs, the travel time of lay first responders is shorter than the travel time of ambulances. Similar results were described by Hatakeyama et al. [41], who developed an app called "AED-SOS." Use of the app significantly reduced the time for recognizing a cardiac arrest to delivering an AED from 202.2 s to 133.6 s, which may contribute to increasing the chances of survival after OHCA [41]. Berglund et al. [42] investigated the impact of sending volunteer first responders via a smartphone app to fetch AEDs from nearby locations in cases of OHCA. The study showed that although the use of AEDs by volunteer first responders did not increase significantly compared with the control, the use of an app to dispatch volunteers had a positive impact on how quickly AEDs were found and used [42].

The findings of Sakai et al. [43] corroborate the relevance of technology in route calculation. The authors developed a mobile web system called "Mobile AED Map," which displays the location of available AEDs. In a controlled simulation, the study compared travel time and distance to retrieve an AED with and without the use of the mobile map. The results indicated that, although the time to recover AEDs was not significantly different, the traveled distance was significantly shorter with the use of the mobile map, suggesting that technological improvements may increase the app’s usefulness in emergency settings [43]. According to Smith et al. [5], such differences may be attributed to the way routes are calculated. When real obstacles of urban areas are taken into account, it is possible to achieve gains in travel time compared with routes drawn exclusively as straight lines over the geographical space.

McLay and Mayorga [44] argued that maximizing response times to 7 and 8 min may maximize patient survival rates. The authors used data from Hanover County, Virginia, to illustrate that response times within these limits are strongly associated with better survival outcomes, especially in rural regions where response times of 9- and 10-min result in more equitable outcomes for patients [44]. In densely populated urban areas, a response time of 8 min is often not associated with a significant improvement in the rates of survival to hospital discharge, suggesting that, for some medical emergencies, quality of response may be more crucial than speed [45].

In the simulation conducted here, there was a significant difference in median travel times between AED retrieval and ambulance response. The travel time was 223 s for AED retrieval and 309 s for ambulances. Thus, use of well-allocated AEDs in an OHCA scenario may provide better coverage and shorter travel time, potentially improving the clinical outcomes of patients. The simulation is very important, given that a delay in CPR onset in cases of OHCA may directly impact the survival and neurological prognosis of patients.

Studies have shown that the chances of survival after a cardiorespiratory arrest decrease by about 7% to 10% for every minute of delay in initiating CPR [45]. Huang et al. [46] showed that the optimal ambulance response time to maximize the chances of survival after OHCA is 6.2 min. This period can be extended to up to 7.2 min in public places or when CPR is performed by a bystander, showing the positive influence of rapid interventions [46]. In areas where AEDs are well distributed and accessible, such as in certain regions of Japan [47], defibrillation carried out by laypeople before the arrival of emergency services can significantly improve OHCA outcomes. Kobayashi et al. demonstrated that the effectiveness of public defibrillation depends substantially on the specific characteristics of each location, such as population density and AED accessibility [47].

Despite the available features, the Local DEA app has some limitations. For example, it requires internet connection to function properly, without which, it becomes unfeasible to calculate routes or locate AEDs. In cases of no internet connection, the app is limited to providing the CPR guide. Furthermore, the connection speed can affect the app’s performance in calculating routes. However, no other factors were identified that could impair the use of Local DEA. The app did not exhibit performance failures and was considered easy to use by experts. In this study, ambulance travel times were calculated based on the assumption that all ambulances depart from a central station. While this approach provides a standardized model for comparison, it does not account for the dynamic nature of real-world ambulance deployment, where vehicles may be distributed across the city and respond to calls from various locations. This limitation highlights the need for future simulations to incorporate real-time GPS data or typical dispatch patterns to provide more accurate predictions of response times. Additionally, the assumed ambulance travel speed was based on an average of 50 km/h; this speed represents typical urban conditions but does not explicitly account for variations such as peak traffic hours or road closures, which could impact actual response times.

Future work will focus on testing the App with lay users to evaluate its usability, accessibility, and effectiveness in real-life scenarios, ensuring it meets the needs of its primary target audience. Further development will include integrating real-time AED registry data and geolocation services to improve functionality, as well as collaborating with municipalities and emergency services to optimize deployment and tailor features to regional needs. Public engagement and education initiatives will be prioritized to build confidence in using the App during emergencies, and longitudinal studies will assess its long-term impact on survival rates in out-of-hospital cardiac arrests, contributing to global efforts to improve outcomes through innovative public health tools.

## Conclusions

The Local DEA app is an innovative and promising tool to reduce mortality from OHCA. Combining geolocation, AED instructions, and initial care guidelines, the app empowers laypeople to act in critical situations, decreasing response time and increasing the chances of patient survival. Combined with the strategic allocation of AEDs, the app can have significant impacts on public health, contributing to the reduction of the number of deaths due to OHCA.

## Supporting information

S1 File Examples of mobile apps that provide interactive maps showing AED locations in the United States and European and Asian countries. (DOCX)

S2 File Copy of CSUQ. (DOCX)
