# Supplementary material for: Local DEA app: Saving lives with accessible and well-located automated external defibrillators
Source: PLoS One. 2025 Feb 26;20(2):e0318065. doi: 10.1371/journal.pone.0318065 (PMC11864529; doi:10.1371/journal.pone.0318065)
Supplement: S1 File — (DOCX) [file pone.0318065.s001.docx]

**Supplementary material 1** Mobile applications showing interactive maps for locating automated external defibrillators (AEDs)

| **Application** | **Features and important points** |
| --- | --- |
| Crowdsav | Developed by Health Visuals, Singapore. The app allows the user to locate the AED that is closest to their current location, as well as the location of a known AED. Users are notified about incidents happening close to them through push notifications and can help others through chat messaging. Access link: <https://crowdsav.com/>. |
| Staying Alive | Developed by Le Bon Samaritain, France. The app provides the location of more than 65,000 defibrillators worldwide and is available in 13 languages. In Brazil, the number of listed AEDs is insufficient to consider the app functional throughout the country. The main features are as follows: users can add, edit, and report nearby defibrillators; practical guide on basic life support; and news about first aid and Staying Alive community statistics. There is also the possibility of locating an AED near or at a specific location. New AEDs can be registered without internet connection, and the server is updated as soon as the connection is reestablished. Access link: <https://play.google.com/store/apps/details?id=com.mobilehealth.cardiac&hl=en_US> |
| PulsePoint AED | Developed by the PulsePoint Foundation, United States. The app is designed as a simple tool for community input to improve the location of AEDs. Users can add AEDs by typing aed.new in the browser. The emergency AED registry hosted by PulsePoint is integrated with leading providers of emergency medical dispatch and equipped with pre-arrival instructions and tactical maps. In addition to AEDs, the application allows the registration of other lifesaving resources, such as bleeding control kits, naloxone, and epinephrine. A companion app named PulsePoint Respond provides information for CPR-trained individuals willing to help during an upcoming cardiac emergency. Access link: <https://play.google.com/store/apps/details/PulsePoint_AED?id=org.pulsepoint.aeds.android&hl=pt&gl=US> |
| Save a Life | The Save a Life Project originated in Geneva, Switzerland. Its main objective is to develop a network of volunteer first responders trained to intervene in out-of-hospital cardiac arrest (OHCA) cases and create a public AED program to enhance the survival of OHCA victims. The application shows AED locations, images from the European Resuscitation Council illustrating basic life support procedures, instructional videos for administering CPR to adults, children, and infants, and a frequently asked questions section. The app is connected to the 144 call center and allows users to alert rescuers registered in Geneva in the case of an OHCA event. Access link: <https://play.google.com/store/apps/details?id=com.dosgroup.momentum_geneve&hl=pt_BR&gl=US> |
| AED Alert | Developed by Stan - Standby to Help, the Netherlands. The app shows the location of AEDs in the user's vicinity and notifies registered volunteers to assist in OHCA cases. There is also a frequently asked questions section on AEDs. Access link: <https://apps.apple.com/us/app/aed-alert/id1419636757> |
| AED Malta | Not-for-profit app that displays a map showing the location of all available AEDs in the Republic of Malta, including the Island of Gozo. Unlike most apps, AED registration is done via email, rather than through the interface. The app includes instructional videos on how to use an AED. Access link: <https://aedmalta.com/> |
| Goodsam | This platform aims to connect people in emergency situation with trained volunteers and health professionals. An emergency alert is sent to nearby community members, by use of Global Positioning System (GPS) signal and allows the localization and communication with the applicant, by chat or video call. It also identifies the best route to incident location and the availability of AEDs on this. This tool has already  been used by organizations to improve assistance to urgent and emergency victims. Access link:<https://www.goodsamapp.org/> |
