# Supplementary figures and images for: Local DEA app: Saving lives with accessible and well-located automated external defibrillators
Source: PLoS One. 2025 Feb 26;20(2):e0318065. doi: 10.1371/journal.pone.0318065 (PMC11864529; doi:10.1371/journal.pone.0318065)

**Supplementary material 2.** Copy of the CSUQ


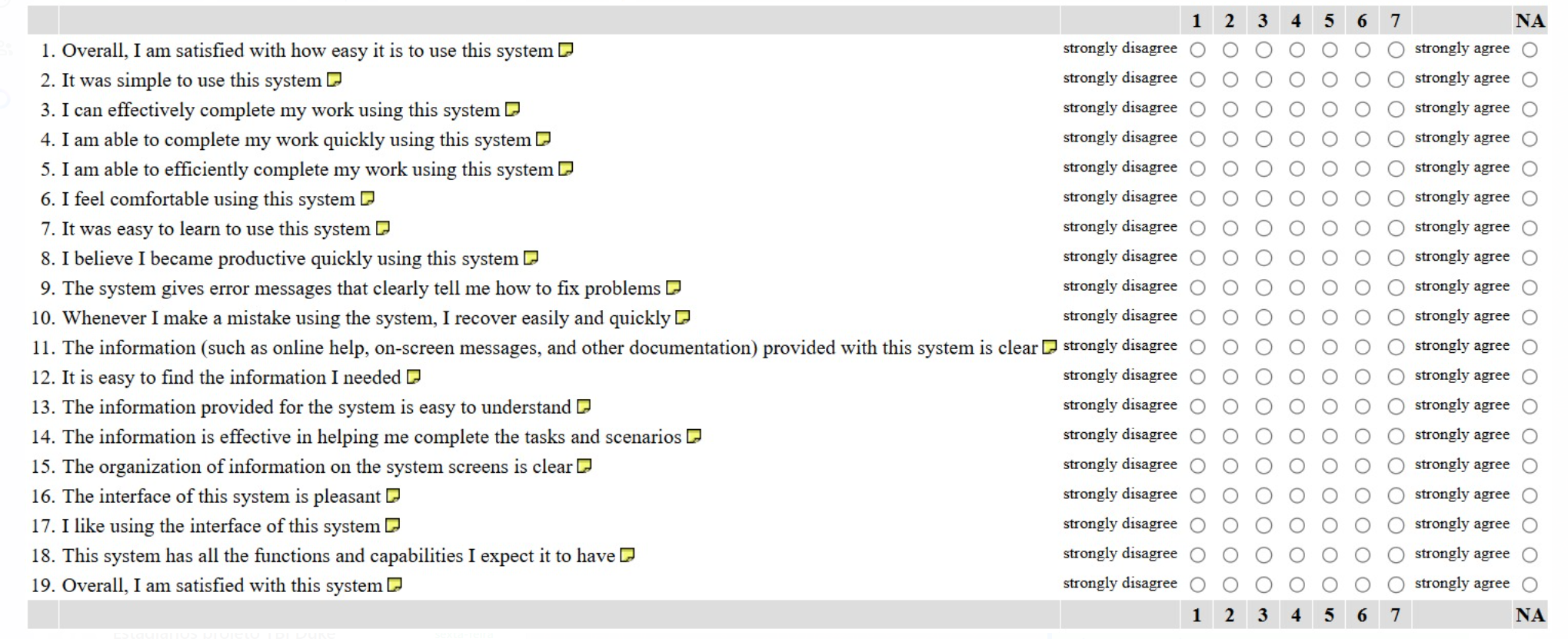

Supplement: S2 File — (DOCX) [file pone.0318065.s002.docx]
